# uPA、ET-1蛋白在非小细胞肺癌中的表达及其临床相关性

**DOI:** 10.3779/j.issn.1009-3419.2011.01.10

**Published:** 2011-01-20

**Authors:** 焱熠 蒋, 一臻 刘, 志周 史, 博石 王, 利 商, 昕 徐, 盛周 张, 明荣 王

**Affiliations:** 1 241000 芜湖，安徽师范大学生命科学学院，生物环境与生态安全省级重点实验室 Key Laboratory of Biotic Environment and Ecological Safety in Anhui Province, College of Life Science, Anhui Normal University, Wuhu 241000, China; 2 100021 北京，中国医学科学院北京协和医学院，肿瘤医院肿瘤研究所分子肿瘤学国家重点实验室 State Key Labouratoy of Molecular Oncology, Cancer Institute (Hospital), Peking Union Medical College and Chinese Academy of Medical Science, Beijing 100021, China

**Keywords:** 肺肿瘤, uPA, ET-1, 免疫组织化学, 预后, Lung neoplasms, uPA, ET-1, Immunohistochemistry, Prognosis

## Abstract

**背景与目的:**

肺癌是世界第一大恶性肿瘤，其发病率及死亡率居高不下，本研究旨在探讨uPA和ET-1蛋白在非小细胞肺癌中的表达状况及其在临床诊断和预后判断方面的应用价值。

**方法:**

采用组织微阵列联合免疫组织化学染色技术，研究155例非小细胞肺癌中uPA和ET-1蛋白的表达情况，分析其与临床病理参数的相关性。

**结果:**

uPA阴性/弱、中度和高表达在鳞癌中的比例分别为12.3%、64.4%、23.3%，在腺癌中分别为12.2%、53.7%、34.1%，在全部病例中分别为12.3%、58.7%、29.0%。ET-1在鳞癌中阴性/弱、中度和高表达分别为2.7%、42.5%、54.8%，在腺癌中分别为11.0%、30.5%、58.5%，在全部病例中分别为7.1%、36.1%、56.8%。uPA和ET-1同时高表达多见于无淋巴结转移的腺癌中（*P*=0.017）。uPA高表达或与ET-1同时高表达的腺癌患者具有较长的术后生存时间（*P*=0.007, *P*=0.016）。

**结论:**

检测uPA和ET-1蛋白表达水平变化可能有助于非小细胞肺癌的预后评估。

## Abstract (en)

**Background and objective:**

uPA and ET-1 proteins have been reported to be up-regulated in some of human cancers. The aim of this study is to investigate the alteration and clinical relevance of uPA and ET-1 protein levels in non-small cell lung cancer (NSCLC).

**Methods:**

Expressions of uPA and ET-1 protein were detected in 155 cases of NSCLC with tissue microarrays and immunohistochemistry (TMA-IHC) technique. The correlations between the alteration of the two proteins and clinicopathological parameters were analyzed.

**Results:**

Negative/weak, moderate and high expression of uPA were observed in 12.3%, 64.4% and 23.3% of squamous cell carcinomas, in 12.2%, 53.7% and 34.1% of adenocarcinomas, and in 12.3%, 58.7% and 29.0% of all cases. ET-1 presented negative/weak, moderate and high expression in 2.7%, 42.5% and 54.8% of squamous cell carcinomas, in 11.0%, 30.5% and 58.5% of adenocarcinomas, and in 7.1%, 36.1% and 56.8% of all cases. Simultaneously high expression of uPA and ET-1 were found in adenocarcinomas without lymph node metastasis (*P*=0.017). Adenocarcinoma patients with high expression of uPA or with high expression of both ET-1 and uPA had the longer survival time (*P*=0.007 and 0.016).

**Conclusion:**

Detection of uPA and ET-1 protein levels might contribute to the prognosis evaluation of NSCLC.

肺癌是世界第一大恶性肿瘤，其发病率及死亡率居高不下，其中80%-85%为非小细胞肺癌（non-small cell lung cancer, NSCLC），预后较差。尽管临床术式和治疗方案不断改进，但肺癌的复发和转移使中晚期NSCLC患者的5年生存率很低。寻找可预测NSCLC患者复发和转移的分子标志物，可能有助于提高临床诊断的精准率，便于提前干预，降低该病的复发和转移。

肿瘤细胞通过血管等发生移位是癌症发生及转移的一个重要途径。尿激酶型血浆纤溶酶原激活因子（urokinase-type plasminogen activator, uPA）是一种重要的蛋白水解酶，可以把纤维蛋白溶酶原转化成纤维蛋白溶酶。uPA通过降解细胞外基质，促使细胞移动。文献^[1-3]^报道uPA蛋白在乳腺癌、宫颈癌和直肠癌等人类恶性肿瘤组织中表达上调。体外研究^[4, 5]^表明uPA在促进肿瘤侵袭转移中起重要作用。

内皮素-1（endothelin-1, ET-1）是一种强效的缩血管肽和血管平滑肌细胞有丝分裂原，ET-1通过自分泌和旁分泌调节作用，促进细胞增殖、浸润以及血管的生成。ET-1蛋白水平在部分恶性肿瘤中是升高的，如膀胱癌^[6]^、结直肠癌^[7]^、前列腺癌^[8]^以及NSCLC^[9]^。卵巢癌的研究^[10]^显示ET-1能加快卵巢癌细胞的运动能力，增强肿瘤的侵袭性和转移。Boldrini等^[11]^报道具有高水平ET-1 mRNA的肺癌组织同时表现ET-1蛋白高表达。

本文通过组织微阵列联合免疫组织化学染色技术（tissue microarrays and immunohistochemistry, TMA-IHC）检测了155例非小细胞肺癌组织标本中的uPA和ET-1蛋白表达，进而分析了与临床、病理参数的相关性，以探讨其临床意义。

## 1 材料与方法

### 1.1 材料

共收集2006年7月-2009年7月中国医学科学院肿瘤医院胸外科肺癌手术标本155例。全部病例均由病理科诊断为NSCLC。其中，鳞癌73例，腺癌82例；男性110例，女性45例；年龄32岁-80岁，中位年龄60岁；肿瘤分期T2、T3、T4期分别为68例、53例和34例，有、无淋巴结转移各为112例和43例。其中有完整随访资料的病例为107例，死亡病例61例，平均随访时间为20个月（2个月-44个月）。

### 1.2 试剂

兔抗人uPA抗体（001098-R/R1，稀释浓度1:200）、兔抗人ET-1抗体（000202-R/R1，稀释浓度1:200）购于赛驰公司，PV-9000免疫组织化学试剂盒和DAB显色试剂盒购自北京中杉金桥生物技术有限公司。

### 1.3 组织芯片制作

常规制作标本蜡块、切片。HE染色后经病理学专家复诊、定位标记。根据定位结果对石蜡块进行打点，每个病例用穿刺针取三个标记好的癌组织点，转移到芯片蜡块的相对应位置，对其进行切片。

### 1.4 免疫组化实验步骤

组织芯片常规脱蜡水化后，3%H_2_O_2_封闭内源性过氧化物酶15 min，0.1%枸橼酸钠缓冲液中微波修复20 min，滴加一抗，4 ℃冰箱过夜，第二天依次加入试剂1和试剂2，在37 ℃下分别孵育20 min和30 min。DAB染色，苏木素复染，梯度酒精脱水，二甲苯透明，中性树胶封片，镜检。

### 1.5 结果判定

以胞浆内出现棕黄色颗粒为阳性信号，以阳性肿瘤细胞百分率和染色强度两方面分别进行半定量评分。染色强度评价方法：不着色为0分，浅棕黄色为1分，中度着色为2分，深棕黄色为3分。阳性肿瘤细胞百分率评价方法：无阳性细胞为0分，阳性肿瘤细胞数1%-33%为1分，阳性肿瘤细胞数34%-67%为2分，阳性肿瘤细胞数达到67%-100%为3分。染色强度和阳性细胞百分率得分相加记为综合分，0分-2分为阴性/弱阳性，3分-5分为中度阳性，6分为强阳性。

### 1.6 统计学分析

使用SPSS 15.0软件进行数据统计，采用双侧卡方检验方法分析蛋白表达改变与临床病理参数的相关性，*Kaplan-Meier*方法分析蛋白表达与患者术后生存之间的关系。*P* < 0.05为有统计学差异。

## 2 结果

### 2.1 uPA和ET-1在NSCLC组织中的表达情况

uPA阴性/弱阳性、中度阳性和强阳性表达在鳞癌中的比例各为12.3%、64.4%和23.3%，在腺癌中分别为12.2%、53.7%、34.1%，在全部病例分别为12.3%、58.7%、29.0%。ET-1在鳞癌中阴性/弱阳性、中度阳性和强阳性表达分别为2.7%、42.5%、54.8%，在腺癌中各为11.0%、30.5%、58.5%，在全部病例分别为7.1%、36.1%、56.8%（表 1，图 1，图 2）。

**1:** 155例NSCLC组织uPA和ET-1表达的免疫组化检测结果 The expression of uPA, ET-1 protein in 155 cases of NSCLC by immunohistochemistry

Protein	Score	SCC			ADC			All cases	
		Cases (*n*=73)	Rate		Cases (*n*=82)	Rate		Cases (*n*=155)	Rate
uPA	0-2	9	12.3%		10	12.2%		19	12.3%
	3-5	47	64.4%		44	53.7%		91	58.7%
	6	17	23.3%		28	34.1%		45	29.0%
ET-1	0-2	2	2.7%		9	11.0%		11	7.1%
	3-5	31	42.5%		25	30.5%		56	36.1%
	6	40	54.8%		48	58.5%		88	56.8%
SCC: squamous cell carcinoma; ADC: adenocarcinoma; NSCLC: non-small cell lung cancer.									

**1:**
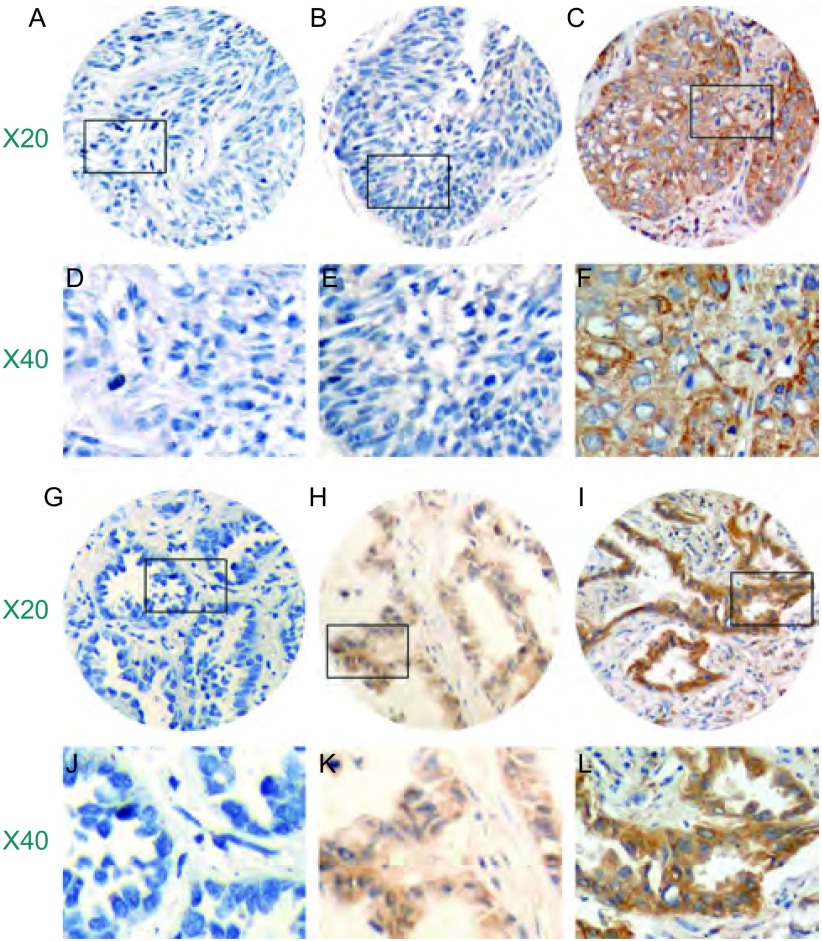
uPA在NSCLC中表达的免疫组化染色 The expression of uPA in NSCLC with immunohistochemistry. Left to right: negative/weak, moderate and high expression; A-F: squamous cell carcinoma; G-L: adenocarcinoma.

**2:**
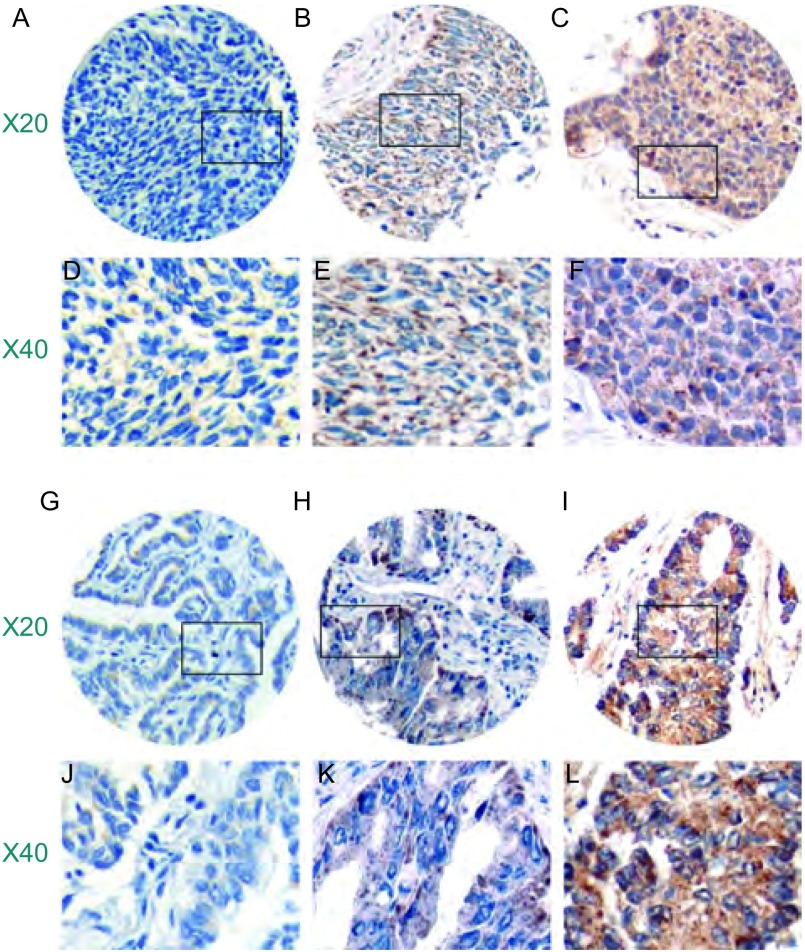
ET-1在NSCLC中表达的免疫组化染色 The expression of ET-1 in NSCLC with immunohistochemistry. Left to right: negative/weak, moderate and high expression; A-F: squamous cell carcinoma; G-L: adenocarcinoma.

### 2.2 uPA和ET-1表达与NSCLC患者临床病理参数的相关性

对uPA、ET-1表达与肿瘤组织的分期、区域淋巴结转移及分化程度进行相关性分析，结果显示单个蛋白在鳞癌的表达与以上临床病理参数均无关。uPA和ET-1同时高表达（强阳性）多见于无淋巴结转移的腺癌中（*P*=0.017）（表 2）。

**2:** uPA和ET-1在腺癌中高表达与临床病理参数的关系 The relationship between uPA, ET-1 protein high xepression and clinicopathological parameters of adenocarcinoma

Clinicopathologic characteristic	*n*	uPA		*P*	ET-1		*P*	uPA and ET-1		*P*
		Positive	Rate		Positive	Rate		Positive	Rate	
TNM stage				0.754			0.940			0.649
T2	43	14	32.6%		25	58.1%		13	30.2%	
T3	20	5	25.0%		9	45.5%		3	15.0%	
T4	19	9	47.4%		14	70.0%		7	36.8%	
Lymph node metastasis^*^				0.053			0.182			0.017
No	25	12	48.00%		17	68.0%		11	44.0%	
Yes	54	12	22.2%		28	51.9%		10	18.5%	
Differentiation				0.191			0.662			0.234
High	8	4	50.0%		5	62.5%		4	50.0%	
Moderate	52	19	36.5%		31	57.4%		15	28.8%	
Low	22	5	22.7%		12	54.5%		4	18.2%	
^*^3 cases of NX were not included.										

### 2.3 uPA和ET-1蛋白表达与NSCLC患者术后生存时间的关系

经*Kaplan-Meier*分析，ET-1表达与NSCLC患者术后生存时间无关，uPA蛋白高表达或与ET-1同时高表达的腺癌患者具有较长的术后生存时间（*P*=0.007, *P*=0.016）（图 3，图4）。

**3:**
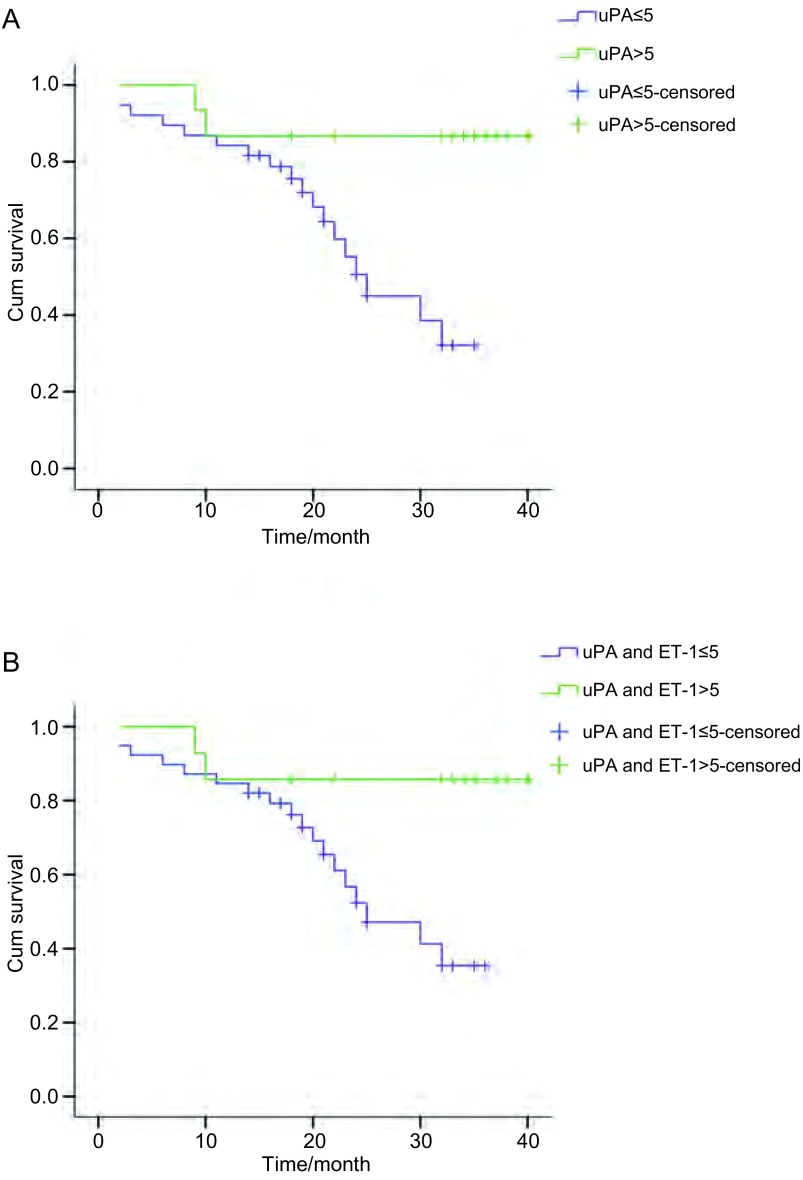
uPA、ET-1表达与腺癌患者术后生存时间相关性的*Kaplan-Meier*分析 The *Kaplan-Meier* survival curves of adenocarcinoma patients with expressions of uPA and ET-1. A: uPA expression (*P*=0.007);B: Expression of both uPA and ET-1 (*P*=0.016).

## 3 讨论

恶性肿瘤由于其细胞生长、增殖失控等多因素、多层面的不确定因素，导致目前在临床上还没有一个很好的治疗办法。肺癌是威胁人类健康最主要的恶性肿瘤之一，其发病率及死亡率均居于癌症榜首。依靠传统的肺癌患者预后指标尚难把不同发病机制的患者区分开来。从分子水平寻找一些敏感性、特异性高的NSCLC相关分子标志物，可能为现有的预后指标提供重要补充。

uPA于1976年被Astedt等^[12]^在卵巢肿瘤细胞中发现。uPA以无活性的单链丝氨酸酶原形式由肿瘤细胞或其它细胞分泌，在细胞表面与uPAR（uPA的受体）结合后可被细胞表面的纤溶酶、舒血管素等激活。促进肿瘤细胞向受破坏的细胞外基质移动，引起肿瘤局部浸润性生长并进入脉管系统，进而发生远处转移^[13]^。本研究发现NSCLC组织中存在uPA蛋白高表达，与国内外在其它肿瘤中观察的uPA变化相符。Yang等^[14]^在结直肠癌研究中发现uPA蛋白升高与淋巴结转移无相关性（*P* > 0.05），也与本实验研究结果类似。Konecny等^[15]^报道uPA蛋白升高与卵巢癌患者较短的生存时间相关，而且可能作为一个独立的预后分子标志物。而本研究结果显示uPA蛋白高表达的肺腺癌患者却具有较长的生存时间，是否uPA在不同癌症中的作用有所差异，值得进一步研究。

内皮素（ET）家族中有三种异构体：ET-1、ET-2和ET-3，三者之间仅有2个-6个氨基酸不同，具有组织特异性^[16]^。人的*ET-1*基因全长12, 464 bp，含有5个外显子。ET-1是体内最强的缩血管活性肽，广泛存在于血管内皮细胞及神经细胞内，在很多肿瘤中都有过度表达。在肿瘤发生发展的过程中，血管生成是一个较早期事件。本研究发现ET-1蛋白在NSCLC组织中表达上调，与文献报道一致。刘玉春等^[9]^采用免疫组织化学方法检测发现，ET-1在NSCLC组织中的表达高于肺良性病变组织，尤其是在具有淋巴结转移阳性的肺癌组织中。我们并没有观察到ET-1与NSCLC的任何临床、病理参数存在相关性。但是，本研究的结果显示uPA和ET-1同时高表达多见于无淋巴结转移的腺癌中，而且uPA和ET-1同时高表达的腺癌患者具有较长的术后生存时间。这一结果也反映了肿瘤发生发展中的多基因异常，uPA和ET-1蛋白的联合检测可能有助于肺腺癌的预后判断。
